# Comparing the Quality of Primary Care Electronic Health Record Data in Australia and Canada: Case Study in Osteoarthritis

**DOI:** 10.2196/69631

**Published:** 2025-07-03

**Authors:** Sharmala Thuraisingam, D Himasara Marasinghe, Kendra Barrick, Fariba Aghajafari, Jo-Anne Manski-Nankervis, Michelle M Dowsey, Hude Quan, Tyler Williamson, Stephanie Garies

**Affiliations:** 1 Department of Surgery University of Melbourne Melbourne Australia; 2 Department of Family Medicine Cumming School of Medicine University of Calgary Calgary, AB Canada; 3 Ridgeview Medical Centre Canmore, AB Canada; 4 Family Medicine and Primary Care Lee Kong Chian School of Medicine Nanyang Technological University Singapore Singapore; 5 Department of Community Health Sciences Cumming School of Medicine University of Calgary Calgary, AB Canada

**Keywords:** electronic medical records, electronic health records, data quality assessment, primary care, general practice, family practice, data linkage, osteoarthritis

## Abstract

**Background:**

General practice electronic health records (EHRs) contain a wealth of patient information. However, these data are collected for clinical purposes. Hence, questions remain around the suitability of using these data for other purposes, including epidemiological research, developing and validating clinical prediction models, conducting audits, and informing policy.

**Objective:**

This study aimed to compare the quality of osteoarthritis-related data in Australian and Canadian general practice EHRs for externally validating a clinical prediction model for total knee replacement surgery.

**Methods:**

A data quality assessment was conducted on 201,462 patient general practice EHRs from Australia provided by National Prescribing Service MedicineWise, and 92,425 from Canada provided by the Canadian Primary Care Sentinel Surveillance Network. Completeness, plausibility, and external validity of data elements relevant to osteoarthritis were assessed. Completeness and plausibility were evaluated using counts and proportions. For external validity, prevalence was estimated using proportions, and knee replacement summarized as a rate per 100,000 population.

**Results:**

There were minimal incomplete and implausible data fields for age and sex (<1%), geographic location (<5%), and commonly cooccurring comorbidities (<10%) in both datasets. However, weight, height, BMI, and Canadian Index of Multiple Deprivation contained >50% missing data. The recording of osteoarthritis by age and sex in both datasets were similar to national estimates, except for patients aged >80 years (Australia: 16.6%, 95% CI 16%-17.3% vs 13.1%, 95% CI 11.2%-15.4%; Canada: 36.7%, 95% CI 36.1%-37.2% vs 50.8%, 95% CI 50.7%-50.9%). Total knee replacement rates were substantially lower in both EHR datasets compared with national estimates (Australia: 72 vs 218 per 100,000; Canada: 0.84 vs 200 per 100,000).

**Conclusions:**

Age, sex, geographic location, commonly cooccurring comorbidities, and prescribing of osteoarthritis medications in Australian and Canadian general practice EHRs are suitable for use in clinical prediction model validation studies. However, BMI and the Canadian Index of Multiple Deprivation are unfit for such use due to large proportions of missing data. Rates of total knee replacement surgery were substantially underreported and should not be used for prediction model validation. Better harmonization of patient data across primary and tertiary care is required to improve the suitability of these data. In the meantime, data linkage with national registries and other health datasets may overcome some of the data quality challenges in general practice EHRs.

## Introduction

### Background

Primary care electronic health records (EHRs) are a rich source of patient data [1,2]. They typically contain administrative and clinical information, including demographics, clinical observations, past and current diagnoses and medications, pathology and imaging results, referral letters to other health care providers, and billing information [2]. Given that patients attend general practice on average 5 times a year [3], data within these records are longitudinal in nature and may allow tracking of a patient’s health journey over time. The large volumes of data contained within primary care EHRs have led to these data being used for a variety of purposes, including disease surveillance, longitudinal studies, prediction modeling, auditing primary care, pharmaco-epidemiological studies, study of rare diseases, and informing policy [2,4,5]. Despite the potential of primary care EHRs to facilitate a variety of secondary purposes, there are challenges with the use of this data source. Data within primary care EHRs are collected for clinical purposes and may therefore be influenced by the processes used to collect, amalgamate, extract, and disseminate the data, which could lead to biased research outcomes [5,6]. For instance, patients who attend general practice frequently are more likely to have complete records, as there is more opportunity for data to be captured by the general practitioner and recorded in the EHR [1]. However, these patients may tend to be sicker or have better access to health care. Moreover, jurisdictions where certain chronic conditions are incentivized by governments may have more consistently recorded information in primary care EHRs compared with unincentivized conditions [6]. These examples demonstrate that missing patient EHRs or clinical information from EHR databases may affect research validity or generalization of findings. The lack of a standardized primary care EHR software system in both Australia and Canada presents further challenges for researchers intending on using these data. Inconsistent data formats and structures require complex data extraction and amalgamation processes which have the potential to introduce bias in the data [6,7]. Structured data fields generally promote completeness and plausibility by guiding data entry through predefined formats, minimizing missing or illogical values [5]. In contrast, free-text data fields allow for more nuanced documentation but can lead to variability, making data harder to standardize, analyze, and code. Relying solely on EHR data from practices that use structured data fields may provide a biased view of the disease under study, in that it may not fully reflect the intent of the author (ie, clinician). Various countries have introduced incentive schemes to improve the quality of data recorded in EHRs [8-11]. While these programs demonstrated improved EHR data accuracy and completeness, and better standardization of recording practices, they also reported challenges in integration with workflow, unrealistic performance metrics, and a focus on meeting numeric targets as opposed to ensuring data accuracy [8-11]. To minimize the likelihood of bias findings in research using EHRs, it has been recommended to first assess the quality of EHR data in the context in which the data will be used [12,13]. The aim is to determine whether these data are suitable for the intended research purpose and to only proceed using these data for research once “fitness for use” has been established [12,13]. Weiskopf and Weng [14] and Kahn et al [15] developed frameworks for assessing the quality of EHR data. The framework developed by Weiskopf and Weng [14] is more foundational and conceptual, focusing on the implications of EHR data quality for research. While Kahn et al [15] provide a practical and standardized approach, tailored for multisite EHR-based research. Both frameworks incorporate similar data quality domains but differ slightly in terminology. Commonly proposed data quality domains include completeness, plausibility, conformance, accuracy, currency, and external validity [14,15]. To date, very few studies using primary care EHRs have published data quality assessments beforehand [16,17]. In recent years, automated data quality assessment programs have gained traction due to their efficiency and scalability [18-20]. However, concerns remain around the accuracy and reliability of these tools, the lack of a standardized data quality assessment framework, and the ability to assess data quality in the context in which the data will be used [18-20].

### Objectives

Our research team had previously conducted a data quality assessment of Australian primary care EHR data in the context of osteoarthritis and developed a clinical prediction model for total knee replacement (TKR) surgery from these data [17,21]. Given the similarities in prevalence and management of osteoarthritis in Australia and Canada [22,23], our prediction model may be applicable to Canadian patients with osteoarthritis. This study aimed to compare the quality of osteoarthritis-related data recorded in Australian and Canadian primary care EHRs using the practical and comprehensive data quality framework proposed by Kahn et al [15]. The purpose was to use Canadian EHR data to externally validate a clinical prediction model for TKR surgery, which was originally developed using Australian EHR data [17,21]. Osteoarthritis is a degenerative joint disease characterized by the breakdown of cartilage between bones [22,23]. It affects 9% of Australians (approximately 2.2 million) and 14% of Canadians (approximately 3.9 million) [22,23]. Osteoarthritis causes pain, inflammation, and physical limitations and can have a substantial impact on a person’s quality of life [23]. Osteoarthritis has been ranked as the 13th leading cause of years lived with disability globally [24]. Given that osteoarthritis is typically diagnosed and managed in community-based settings, primary care EHR data is an ideal source for osteoarthritis research and surveillance.

## Methods

### Data Sources

#### Australian Data Source

National Prescribing Service (NPS) MedicineWise is an Australian not-for-profit organization focusing on the improvement of health through the appropriate use of medications and health technologies [25]. They manage the MedicineInsight program which aims to identify key areas for improvement in primary care using data from consenting general practices across Australia [26]. The MedicineInsight dataset contains deidentified EHRs from over 2.9 million patients from 671 consenting general practices around Australia [26,27]. Third party data extraction tools are used to deidentify, extract, and securely transmit patient data monthly from general practice clinics to a central data warehouse [26]. Here, structured data from 2 different EHR software systems, Medical Director and Best Practice [28,29], were merged into a consistent format before being provided to the researcher [26,30]. The MedicineInsight data includes patient demographics, medications, diagnoses, procedures, clinical observations, pathology, allergy, and alcohol status. Data fields containing raw text were provided as is, unless otherwise requested by the researcher. Medical Director uses the Docle diagnosis coding system and Best Practice uses Pyefinch [26]. Clinicians do not always need to use a coded diagnosis and can enter diagnoses as free text. Patient progress notes were not provided to researchers due to this field potentially containing patient identifying information.

#### Canadian Data Source

The Canadian Primary Care Sentinel Surveillance Network (CPCSSN) is a national collaboration of practice-based research networks collecting deidentified EHR data from contributing primary care physicians and nurse practitioners [31,32]. The data are extracted, cleaned, and processed biannually by each regional practice-based research network, then merged in a central repository located at Queen’s University in Kingston, Ontario. The 2018 national CPCSSN database included clinical information from nearly 1.8 million patients and over 1200 health care providers from 251 practices [31]. The CPCSSN data contain most patient information from the EHR, including demographics, diagnoses (current and historic), prescribed medications, physical examinations (height, weight, BMI, and blood pressure), laboratory results, referrals, risk factors, vaccinations, and allergies [32]. In Canadian primary care settings, the International Classification of Disease version 9 is the standard system used for coding diagnoses and billing claims, though free-text words also are used throughout the EHR [33].

### Ethical Considerations

Ethics approval was provided by the University of Melbourne Human Research Ethics Committee (ID 1852593) and the University of Calgary Conjoint Health Ethics Board (REB20-0213) in September 2018. This study was also approved by the NPS MedicineWise data governance committee in July 2021.

### Study Sample

#### Australian Study Sample

NPS MedicineWise provided data extracted from 475,870 patient EHRs with a recorded diagnosis of osteoarthritis from 483 Australian general practice clinics. The coding used by NPS MedicineWise to identify patients with osteoarthritis has been provided in Multimedia Appendix 1. Patient sociodemographic and clinical data recorded in the EHRs were extracted up until December 31, 2017 (inclusive) and encounter data between the years 2013 to 2017 (inclusive). Clinical data included clinical observations, prescribed medications, pathology results, diagnoses, and medical procedures.

Study baseline was defined as January 1, 2014. Patients were included in the study if they had attended general practice at least once in the year before baseline (2013) and were aged ≥45 years.

#### Canadian Study Sample

**Figure 1:**
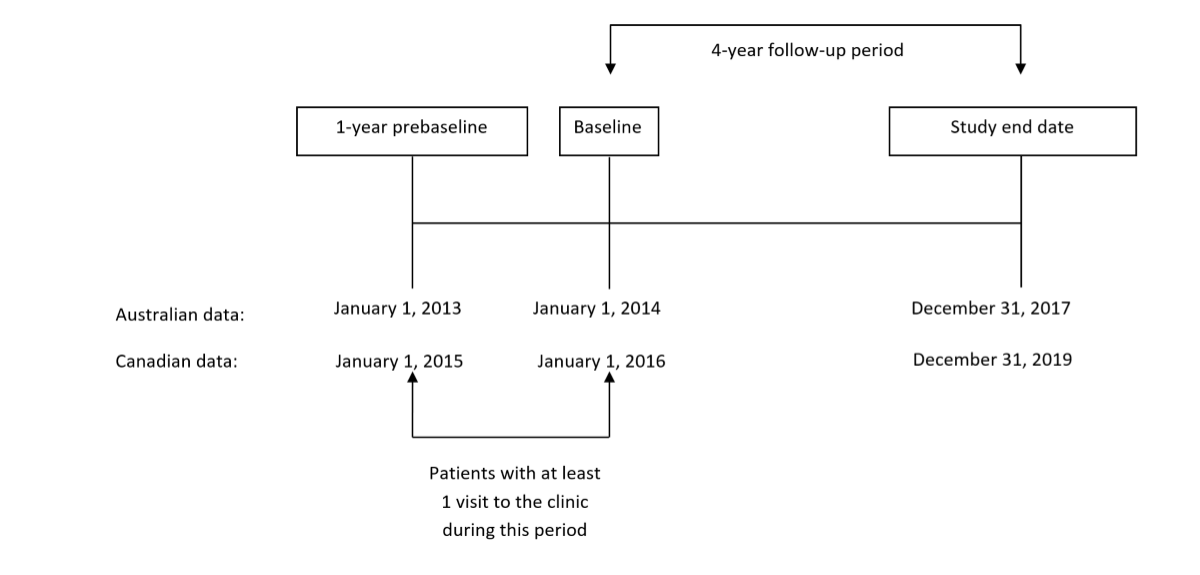
Study timeline for Australian and Canadian datasets.

Patient data were extracted up until December 31, 2019 (inclusive). The CPCSSN definition for osteoarthritis was used to identify patients with osteoarthritis [34]. The definition uses *International Classification of Diseases, Ninth Revision* codes (715, 721) found in the billing table or the problem list or profile table (at least one at any time) to index a patient with osteoarthritis. When validated against medical chart review as the reference standard, the CPCSSN case definition showed reasonable accuracy with a sensitivity of 77.8%, specificity 94.9%, positive predictive value 87.7%, and negative predictive value 90.2% [34]. Multimedia Appendix 2 [34-36] provides the definitions for osteoarthritis within the CPCSSN database. Study baseline was defined as January 1, 2016, for the Canadian cohort. Patients who were ≥45 years with at least one encounter with a primary care provider in the year before baseline (2015) were included in this study. Data from the encounter, billing, medications, or diagnosis EHR tables were used to identify patient encounters. Patients assigned an “inactive” EHR status by their individual clinic or a “deceased” status with a date before baseline (2015) were removed from the analysis. Figure 1 illustrates the study timelines for the Australian and Canadian datasets.

### Coding of Variables

#### Coding of Variables in Australian Data

All sociodemographics, clinical observations, and comorbidities were coded from the Australian EHRs on January 1, 2014. Geographic location of the patient and general practice was categorized using the Australian Statistical Geography Standard remoteness areas of the Australian Bureau of Statistics [37]: major cities of Australia, inner regional Australia, outer regional Australia, remote Australia, and very remote Australia. These categories were dichotomized into urban and rural with remote and very remote Australia defined as rural. The Australian Index of Relative Socioeconomic Advantage and Disadvantage was coded by NPS MedicineWise using patient postcodes. These data were provided in quintiles with 1 representing those most disadvantaged and 5 representing those most advantaged.

The prevalence of commonly cooccurring chronic conditions in patients with osteoarthritis were summarized [23]. These included hypertension, lipid disorder, ischemic heart disease, depression, anxiety, asthma, diabetes mellitus, chronic obstructive pulmonary disease, and metastatic solid tumor. The prevalence of chronic conditions listed in the Charlson Comorbidity Index were also summarized [38]. The diagnosis onset date was used to determine which patients had a recorded diagnosis of these conditions in their EHR at study baseline. The coding used to identify patients with these chronic conditions is listed in Multimedia Appendix 1. For clinical observations, the latest weight, height, and BMI recorded in the EHRs in the year before baseline was extracted.

Osteoarthritis medications were defined as medications belonging to the following Anatomical Therapeutic Code classes: H02—corticosteroids for systemic use, M01—anti-inflammatory and antirheumatic products, M02—topical products for joint and muscular pain, M09—other drugs for disorders of the musculoskeletal system, N01—anesthetics, N02—analgesics, and N06—psychoanaleptics. The strength, dosage, and frequency fields of the prescription data were used to calculate whether patients were likely to be taking osteoarthritis medications in the year before study baseline using prescriptions issued in the 12 months before baseline. Patients with prescriptions with missing strengths, dosages, or frequencies were coded as having missing data for that medication as it was not possible to determine whether the patient was likely to be taking that medication at study baseline. Multimedia Appendix 1 contains a full list of the osteoarthritis medications included in this study.

#### Coding of Variables in Canadian Data

All sociodemographics, clinical observations, and comorbidities were included in the analysis from the Canadian EHRs as of January 1, 2016. Coding of variables in the Canadian EHR data was similar to the Australian data, except for geographic location of the patient, socioeconomic status, and prescribing of osteoarthritis medications. Here, slight differences existed in the coding of variables due to differences in how these data were recorded in the Canadian and Australian EHR systems. The Canadian postal code was used to determine the geographic location of the patient. Postal codes that include a “0” as the second character were coded as rural delivery areas and the remaining as urban [39]. Information from the Canadian Population Census was used to derive indicators for deprivation: residential instability, economic dependency, ethno-cultural composition, and situational vulnerability, at a dissemination area level in the Canadian Index of Multiple Deprivation. The Postal Code Conversion File Plus [40] was used to assign a dissemination area and thus a quintile ranking of deprivation for each of the 4 dimensions of the Canadian Index of Multiple Deprivation to patients based on their recorded postcode in the EHR. The index was averaged across these 4 dimensions to obtain a composite index for socioeconomic status where 1 represented least deprived and 5 represented most deprived.

Prescriptions were considered in use at study baseline if a patient had at least one record of a given osteoarthritis medication with a prescription start date within the prebaseline period. EHR records with a missing start date of prescription were considered as having missing data. Multimedia Appendix 2 contain further information on how Canadian variables were coded for this analysis.

### Data Quality Assessment and Statistical Methods

#### Overview

The quality of data in Australian and Canadian primary care EHRs was assessed using the data quality framework proposed by Kahn et al [15]. Data fields selected for assessment were those related to predictors from the clinical prediction model [21] and variables relevant to osteoarthritis as determined through a literature search and an adapted Delphi process involving experts in the field of osteoarthritis [41]. Three domains of data quality were assessed where possible as follows: (1) incompleteness, (2) implausibility, and (3) external validity. The remaining data quality domains from the Kahn et al [15] framework could not be assessed due to limitations in the availability of EHR data fields and the absence of external gold-standard data sources for verification and validation.

#### Incomplete and Implausible Data

Incompleteness was assessed by considering the context in which the data were collected and the management of osteoarthritis in general practice. For example, weight is typically entered in the clinical observations field in Australian general practice EHRs and in an examination record in Canadian general practice EHRs. Hence, the proportion of patients without a recorded weight observation (Australian data) or weight examination record (Canadian data) in the year before baseline were summarized in addition to those with incomplete data in their clinical observation or examination record. Definitions for implausible data entries are listed in Table 1. Incomplete and implausible data were summarized using counts and percentages.

**Table 1:** Implausible data entry definitions.

Variable	Definition of implausible data entry	
	Australian EHR^a^ data	Canadian EHR data
Age at study start	Year of birth beyond data extraction end date of December 31, 2017	Year of birth beyond data extraction end date of December 31, 2019
Height (cm)	<100 or >240	<120 or >200
Weight (kg)	<20 or >280	<20 or >280
BMI (kg/m^2^)	<12 or >90	<12 or >90
Previous or contralateral TKR^b^	Before January 1, 1960, or the year of surgery was before the year of birth	Before January 1, 1960, or the year of surgery was before the year of birth
Any past knee surgery	Before January 1, 1960, or the year of surgery was before the year of birth	Before January 1, 1960, or the year of surgery was before the year of birth
TKR	Before January 1, 1960, or the year of surgery was before the year of birth	Before January 1, 1960, or the year of surgery was before the year of birth
OA^c^ medications	Prescription date before January 1, 1990	Prescription date before January 1, 1990
Death	Before year of birth	Before year of birth

^a^EHR: electronic health record.

^b^TKR: total knee replacement.

^c^OA: osteoarthritis.

#### External Validity

External validity of the prevalence of osteoarthritis in the Canadian EHRs by age and sex were summarized and compared to national data from the Canadian Chronic Disease Surveillance System (CCDSS) 2018 [42]. Estimates were standardized by age and sex using the Canadian 2021 Census population as the reference standard [43]. Equivalent comparisons were unable to be conducted in the Australian EHRs due to limited access to EHRs from patients without a diagnosis of osteoarthritis. Instead, the recording of osteoarthritis in the Australian EHRs was summarized by age and sex and estimates compared with data from the Australian Bureau of Statistics 2014 to 2015 National Health Survey [44]. The proportions from the National Health Survey were adjusted to account for the survey sampling strategy [45]. The SEs of the proportions were estimated using method 2 of the recommended approaches by Donath [45] for analyzing National Health Survey data. Here, each of the 30 replicate weights provided in the National Health Survey dataset were used to estimate the SEs and hence 95% CIs for the prevalence estimates [45].

The recorded rates of TKR surgery (number of surgeries divided by number of patients) in the EHRs in the 4-year follow-up period were compared with national estimates from the Australian Institute of Health and Welfare National Hospital Morbidity Database [23] and the Canadian Joint Replacement Registry [46]. Rates of TKR surgery were presented per 100,000 people.

Clinical observations, such as weight, height, and BMI were summarized using means and SDs.

## Results

### Study Cohorts

Selection of the Australian general practice EHR study cohort has been described elsewhere [17,21]. In brief, 475,870 patient EHRs with a recorded diagnosis of osteoarthritis were identified from the Australian MedicineInsight data set. Of these, 236,412 (49.7%) patient EHRs had a general practice encounter in the year before study baseline. A further 34,950 (7.3%) patient EHRs with an encounter recorded in the year before baseline were excluded: 28,069 (5.9%) were aged >45 years, 2117 (0.4%) had died before baseline, and 4764 (1%) underwent bilateral TKR before study baseline. A total of 201,462 (42.3%) Australian general practice EHRs were available for analysis.

From the CPCSSN EHR database, 123,741 patient EHRs with a recorded diagnosis of osteoarthritis were identified. A further 10,141 (8.2%) patient EHRs were excluded due to their inactive status, 19,631 (15.9%) patients did not have a record of attending general practice in the year before baseline, 1538 (1.2%) patients were younger than 45 years at baseline and 6 (0.005%) had died before baseline. This resulted in a total of 92,425 (74.7%) Canadian general practice patient EHRs for data quality assessment.

### Incomplete or Missing Data

The frequency and proportion of incomplete data fields are summarized in Tables 2-6. There was minimal missing data for age, sex, and geographic location in both the Australian and Canadian EHRs (Table 2). Just over half (48,854/92,425, 52.9%) of the Canadian study population had missing Canadian Index of Multiple Deprivation data due to missing or incomplete postal code information. There were higher proportions of patients with missing dates of diagnosis for hypertension (19,037/201,462, 9.5% vs 36/92,425, 0.04%), lipid disorder (14,192/201,462, 7% vs 76/92,425, 0.08%), depression (19,393/201,462 9.6% vs 25/92,425, 0.03%), and anxiety (35,644/201,462, 17.7% vs 2771/92,425, 3%) in the Australian EHRs compared with the Canadian EHRs. The proportion of patients without an observation or examination record for height (129,552/201,462, 64.3% vs 52,867/92,425, 57.2%), weight (112,230/201,462, 55.7% vs 53,654/92,425, 58.1%) and BMI (137,071/201,462, 68% vs 50,803/92,425, 55%) in the year before study baseline was substantial in the 2 cohorts, and slightly higher for height and BMI in the Australian EHR data compared with the Canadian EHR data (Table 4). In total, >1% of patients in the Canadian dataset had a procedure record relating to knee surgery (including TKR surgery) before and during the 4-year study period (Table 5). In comparison, this was approximately 10% in the Australian cohort. There were higher proportions of missing data in osteoarthritis medication records in the Australian EHR data compared with the Canadian data (Table 6). However, different approaches were used to classify missing data in osteoarthritis medication records between the datasets. In the Australian dataset, missing osteoarthritis medication data could be due to missing medication dosage, strength, or frequency. These fields were not available in the Canadian dataset and therefore only missing prescription dates were considered.

**Table 2:** Missing or incomplete demographics, death, and Charlson Comorbidity Index (CCI data in Australian and Canadian primary electronic health records (EHRs). The mean age was 67.2 (SD 11.1) years in the Australian data and 67.6 (SD 11.4) years in the Canadian data.

Characteristics					Australian EHRs (MedicineInsight data; n=201,462), n (%)		Canadian EHRs (CPCSSN^a^ data; n=92,425), n (%)	
**Demographics**								
	**Age**							
			45-49 years	11,477 (5.7)		4293 (4.6)		
			50-64 years	72,300 (35.9)		34,462 (37.3)		
			65-79 years	84,152 (41.8)		37,926 (41)		
			≥80 years	33,530 (16.6)		15,744 (17)		
			*Missing data*	*3 (0.001)*		*0 (0)*		
	**Sex**							
			Female	123,376 (61.2)		57,157 (61.8)		
			Male	78,049 (38.7)		35,255 (38.1)		
			Other	37 (0.02)		—^b^		
			*Missing data*	*0 (0)*		*13 (0.01)*		
**Geographic location**								
	Urban				173,296 (86)		70,023 (79.1)	
	Rural				27,098 (13.5)		18,557 (20.1)	
	*Missing data*				*1068 (0.5)*		*3845 (4.2)*	
**Socioeconomic status**								
	**Australian index of relative socioeconomic advantage and disadvantage**							
			1 (most disadvantaged)	41,600 (20.8)		N/A^c^		
			2	39,539 (19.8)		N/A		
			3	48,122 (24.0)		N/A		
			4 and 5 (most advantaged)	70,973 (35.5)		N/A		
			*Missing data^d^*	*1228 (0.6)*		*N/A*		
	**Canadian index of multiple deprivation**							
			1 (least deprived)	N/A		628 (1.4)		
			2	N/A		15,750 (36.1)		
			3	N/A		15,666 (36.0)		
			4	N/A		11,210 (25.7)		
			5 (most deprived)	N/A		317 (0.7)		
			*Missing data* ^e^	*N/A*		*48,854 (52.9)*		
**Death**								
		Death during 4-y study period			7720 (3.9)		1436 (1.6)	
		*Missing date of death*			*1861 (0.9)*		*2888 (3.1)*	
**Count of chronic conditions from the CCI^f^**								
		No conditions			113,697 (61.3)		50,131 (46.2)	
		1 condition			49,216 (26.5)		26,510 (29.1)	
		2 conditions			16,407 (8.8)		9777 (10.7)	
		≥3 conditions			6267 (3.4)		4637 (5.1)	
		*Missing date of diagnosis for at least one Charlson comorbidity*			*15,875 (7.9)*		*1370 (1.5)*	

^a^CPCSSN: Canadian Primary Care Sentinel Surveillance Network.

^b^Not available; the other sex category is not recorded in Canadian EHR data.

^c^N/A: not applicable.

^d^It is unknown whether missing data represent patients without a postcode or whether these patients live in low-population areas that do not have an allocated socioeconomic status.

^e^Missing data included missing postal code information in the CPCSSN (n=3845) and incomplete information arising due to CPCSSN data with only first 3 digits of the postal code (n=42,300) and due to lack of assigned deprivation quantities for a subset of dissemination areas when a complete CPCSSN postal code was available (n=2709).

^f^Count of chronic conditions from the CCI for both Australian and Canadian EHRs had a median value of 0 (IQR 0-1).

**Table 3:** Missing and incomplete comorbidity data in Australian and Canadian primary electronic health records (EHRs).

Comorbidity data		Australian EHRs (MedicineInsight data; n=201,462)			Canadian EHRs (CPCSSN^a^ data; n=92,425)	
		Records, n (%)^b^	Missing date of diagnosis, n (%)	Records, n (%)^b^		Missing date of diagnosis, n (%)
**Comorbidities, n (%)**						
	Hypertension	81,004 (44.4)	19,037 (9.5)	38,944 (42.1)		36 (0.04)
	Lipid disorder	58,478 (31.2)	14,192 (7)	54,922 (59.5)		76 (0.08)
	Ischemic heart disease	23,522 (11.8)	2810 (1.4)	10,272 (11.1)		2603 (2.8)
	Depression	35,644 (19.6)	19,393 (9.6)	22,337 (24.2)		25 (0.03)
	Anxiety	16,373 (9.9)	35,644 (17.7)	18,219 (20.3)		2771 (3)
	Asthma	21,757 (11.2)	7088 (3.5)	8715 (9.4)		2421 (2.6)
	Diabetes mellitus	27,821 (14.1)	4205 (2.1)	13,768 (14.9)		75 (0.08)
	Chronic obstructive pulmonary disease	12,709 (6.4)	2977 (1.5)	7410 (8)		15 (0.02)
	Metastatic solid tumor	33,485 (16.9)	3470 (1.7)	334 (0.4)		108 (0.1)

^a^CPCSSN: Canadian Primary Care Sentinel Surveillance Network.

^b^Percentages in these columns are calculated with a denominator representing the total value (in the column header) minus the number of records with missing data.

**Table 4:** Missing or incomplete clinical observation data in Australian and Canadian primary electronic health records (EHRs).

Clinical observations	Australian EHRs (MedicineInsight data; n=201,462)			Canadian EHRs (CPCSSN^a^ data; n=92,425)		
	Values, mean (SD)	Missing data in observation record, n (%)	Missing observation record, n (%)	Values, mean (SD)	Missing a measurement in examination record, n (%)	Missing examination record, n (%)
Height (cm)	165.3 (9.9)	0 (0)	129,552 (64.3)	165.7 (9.9)	1254 (1.4)	51,613 (55.8)
Weight (kg)	82.1 (20.1)	0 (0)	112,230 (55.7)	84.6 (24.6)	9007 (9.7)	44,647 (48.3)
BMI (kg/m^2^)	30.1 (6.5)	0 (0)	137,071 (68)	30.5 (7.4)	270 (0.3)	50,533 (54.7)

^a^CPCSSN: Canadian Primary Care Sentinel Surveillance Network.

**Table 5:** Missing or incomplete knee surgery data in Australian and Canadian primary electronic health records (EHRs).

Knee surgery		Australian EHRs (MedicineInsight data; n=201,462), n (%)	Canadian EHRs (CPCSSN^a^ data; n=92,425), n (%)
**TKR^b^**			
	TKR before study	9432 (4.7)	16 (0.02)
	TKR during 4-y study period^c^	8638 (4.3)	27 (0.03)
	Date missing from knee procedure entry in diagnosis record	1254 (0.6)	121 (0.1)
	Missing knee procedure entry in diagnosis record	182,128 (90.4)	92,261 (99.8)
**Other knee surgery**			
	Past knee surgery (excluding TKR)	6070 (3)	76 (0.08)
	Date missing from knee procedure entry in diagnosis record	990 (0.5)	28 (0.03)
	Missing knee procedure entry in diagnosis record	194,385 (96.5)	92,252 (99.8)

^a^CPCSSN: Canadian Primary Care Sentinel Surveillance Network.

^b^TKR: total knee replacement.

^c^Total knee replacements occurring between 2014 and 2017 for Australian data and between 2016 and 2019 for Canadian data.

**Table 6:** Missing or incomplete osteoarthritis (OA) medication data in Australian and Canadian primary electronic health records (EHRs).

OA medications prescribed		Australian EHRs (MedicineInsight data; n=201,462)		Canadian EHRs (CPCSSN^a^ data; n=92,425), n (%)
		Records, n (%)^b^	Missing data in medication^c^ records, n (%)	
Prescribed ≥1 OA medication in last year^d^		57,090 (33.8)	32,548 (16.2)	44,289 (47.9)
**OA medications prescribed in last year**				
	H02 corticosteroids for systemic use	3457 (1.9)	22,188 (11)	5348 (5.8)
	M01 anti-inflammatory and antirheumatic	21,220 (10.9)	5834 (2.9)	17,500 (18.9)
	M02 topical for joint and muscular pain	0 (0)	387 (0.2)	5412 (5.9)
	M09 other drugs for disorders of musculoskeletal system	0 (0)	58 (0.03)	0 (0)
	N01 anesthetics	0 (0)	34 (0.02)	111 (0.1)
	N02 analgesics	39,882 (22.3)	22,227 (11.0)	20,660 (22.4)
	N06 psychoanaleptics	1987 (1.0)	173 (0.09)	18,998 (20.6)

^a^CPCSSN: Canadian Primary Care Sentinel Surveillance Network.

^b^Percentages in these columns are calculated with a denominator representing the total value (in the column header) minus the number of records with missing data.

^c^Missing data included absence of medication dosage, strength, or frequency.

^d^There were 715 (0.8%) missing medication records and 51 (0.06%) medication records with missing data (including the absence of a medication code or a start date) in the CPCSSN data.

^f^N/A: not applicable.

### Implausible Data

Overall, there were minimal implausible data entries (Table 7) for the data fields under study.

**Table 7:** Implausible data entries in Australian and Canadian primary care electronic health records (EHRs).

Implausible entries	Australian EHRs (MedicineInsight data, n=201,462), n (%)	Canadian EHRs (CPCSSN^a^ data, n=92,425, n (%)
Height	0 (0)	86 (0.1)
Weight	0 (0)	71 (0.1)
BMI	224 (0.11)	96 (0.1)
At least 1 OA^b^ medication in the last year	43 (0.02)	0 (0)
TKR^c^	10 (0.005)	0 (0)
Past knee surgery (other than TKR)	17 (0.01)	0 (0)

^a^CPCSSN: Canadian Primary Care Sentinel Surveillance Network.

^b^OA: osteoarthritis.

^c^TKR: total knee replacement.

### External Validity

Osteoarthritis by age and sex (Table 8) in the Australian EHR data were comparable with national estimates, except for females aged ≥80 years, where proportions were slightly higher in the EHR data compared with the National Health Survey. Age and sex standardized prevalence of osteoarthritis recorded in the Canadian EHRs (Table 9) were comparable with estimates from the CCDSS, except for the ≥80 years age group where prevalence was lower in the EHRs (36.7%, 95% CI 36.1-37.2 vs 50.8%, 95% CI 50.7-50.9).

**Table 8:** External validity of recording of osteoarthritis (OA) in Australian primary care electronic health records (EHRs).

Age categories (y)	OA percentage by age and sex in Australian EHR data (n=201,462)			OA percentage by age and sex in Australian National Health Survey (2014-2015^a^; n=1,933,849)	
	Female, % (95% CI)	Male, % (95% CI)	Female, % (95% CI)		Male, % (95% CI)
45-49	5.8 (5.5-6.1)	5.5 (5.2-5.8)	5.3 (3.9-7.2)		6.3 (4.2-9.3)
50-64	35.7 (34.8-36.5)	36.2 (35.4-37.1)	38.9 (35.9-42)		42.7 (37.4-48.2)
65-79	40.8 (40.1-41.5)	43.3 (42.6-44)	41.8 (39.1-44.6)		39.3 (34.5-44.4)
≥80	17.7 (17.0-18.4)	15 (14.3-15.6)	13.9 (11.5-16.8)		11.6 (8.7-15.3)

^a^From Australian Bureau of Statistics National Health Survey 2014 to 2015 [44].

**Table 9:** External validity of recording of osteoarthritis (OA) in Canadian primary care electronic health records (EHRs).

Age (y)	Prevalence percentage of OA in Canadian EHR data (n=433,474)			Prevalence percentage of OA in Canadian chronic disease surveillance system (2018^a^; n=21,480,750)	
	Female, % (95% CI)	Male, % (95% CI)	Female, % (95% CI)		Male, % (95% CI)
45-49^b^	7.7 (7.4-8)	6.8 (6.5-7.2)	4.2 (4.2-4.2)		3.7 (3.6-3.7)
50-64	18.5 (18.3-18.8)	15.1 (14.9-15.4)	17.8 (17.8-17.8)		13.4 (13.3-13.4)
65-79	33.0 (32.5-33.4)	24.9 (24.5-25.3)	38.3 (38.3-38.4)		27.6 (27.6-27.7)
≥80	39.9 (39.1-40.7)	31.7 (30.9-32.6)	55.9 (55.7-56)		43.2 (43.1-43.4)
Overall^c^	25.0 (24.8-25.2)	19.0 (18.8-19.2)	28.5 (28.5-28.5)		20 (19.9-20)

^a^From Canadian Chronic Disease Surveillance System 2016 [42].

^b^OA prevalence for 45 to 49 age category for Canadian Chronic Disease Surveillance System also includes data for those who were 35 to 44.

^c^Age and sex standardized ratios using the Canadian 2021 Census population as the reference standard [43].

TKR rates recorded in the Australian and Canadian EHRs over the 4-year study period were lower than their respective national estimates (Australian data: 72 per 100,000 EHR vs 218 per 100,000 national estimates; Canadian data: 0.84 per 100,000 EHR vs 200 per 100,000 national estimates).

### Comparing the Characteristics Recorded in General Practice EHRs in Patients With Osteoarthritis From Australia and Canada

Sociodemographic characteristics were similar between the Australian and Canadian cohorts, except a higher proportion of patients in the Australian cohort were from urban areas (173,296/200,394, 86.5% vs 70,023/88,580, 79.1%). Socioeconomic status was not compared between the 2 countries due to high proportions of missing data for the Canadian Index of Multiple Deprivation and differences in the definitions of the measures. A lower proportion of patients had a recorded diagnosis of lipid disorder (58,478/187,270, 31.2% vs 54,922/92,349, 59.5%) and anxiety (16,373/165,818, 9.9% vs 18,219/89,654, 20.3%) in the Australian cohort compared with the Canadian cohort. A higher proportion of patients had a recorded diagnosis of metastatic solid tumor (33,485/197,992, 16.9% vs 334/92,317, 0.4%) in the Australian EHR data compared with the Canadian EHR data. Just over 60% (113,697/185,587) of patients in the Australian EHR dataset did not have a recorded diagnosis of a chronic condition listed in the Charlson Comorbidity Index compared with approximately 55% (50,131/91,055) in the Canadian dataset. Despite substantial amounts of missing data, weight, height, and BMI were similar between the 2 cohorts, and the proportion of patients who died during the 4-year study follow-up was similar. A slightly higher proportion of patients had knee surgery (previous TKR: 9432/200,208, 4.7% vs 16/92,304, 0.02%; TKR during study: 8638/200,208, 4.3% vs 27/92,304, 0.03%; past knee surgery: 6070/200,208, 3% vs 76/92,304, 0.08%) recorded in the Australian EHR data compared with the Canadian data, and a lower proportion of Australian patients (57,090/168,914, 33.8% vs 44,289/92,374, 47.9%) were prescribed at least one osteoarthritis medication in the year before baseline. More specifically, lower proportions of prescribing of anti-inflammatory and antirheumatic products (21,220/195,628, 10.9% vs 17,500/92,374, 18.9%) and psychoanaleptics (1987/201,289, 1% vs 18,998/92,374, 20.6%) were recorded in the Australian EHR data compared to the Canadian EHR data.

## Discussion

### Suitability of EHR Data for Clinical Prediction Model Validation

This study compared the quality of osteoarthritis-related data recorded in Australian and Canadian general practice EHRs for the purposes of externally validating an Australian clinical prediction model for knee replacement surgery using Canadian EHR data. More specifically, the completeness, plausibility, and external validity of data fields relating to osteoarthritis were assessed and compared. Overall, the quality of data recorded in the Australian and Canadian EHRs was similar. Missing data were minimal for all sociodemographic characteristics of interest, except for socioeconomic status in the Canadian data (Canadian Index of Multiple Deprivation). Here data were missing due to patient EHRs containing only the first 3 digits of the postal code (n=42,300, 45.8%) or were missing the postal code (n=3845, 4.2%) or because there was a lack of assigned deprivation quantities for a subset of postal areas (n=2709, 2.9%). While full postal codes are usually collected at the point of care, this is often considered identifiable patient information and is generally not permitted to be extracted and used for secondary data analysis; thus, the CPCSSN research database has a higher proportion of missing full postal code data, and this field may not be useful for Canadian research studies as it currently exists. Further work is needed to understand whether missing Canadian Index of Multiple Deprivation data are likely to be missing completely at random or whether imputation methods for missing data that are missing at random may be used to recover missing deprivation values [47,48].

Similarly, there were substantial missing data for weight, height, and BMI in both the Australian and Canadian EHRs. BMI may be an indicator of osteoarthritis disease progression, and therefore a potentially important data field for osteoarthritis research [41]. Results from this assessment suggest that weight, height, and BMI extracted from EHRs from patients with osteoarthritis may be unsuitable for research use due to large amounts of missing data. Using EHRs with complete BMI data only may lead to biased results; for example, patients who attend general practice more often tend to have more complete EHRs but tend to be in poorer health [1]. In this scenario, using data from patients with complete EHR data only may lead to misrepresentation of the study population of interest as healthier patients are likely to be underrepresented in the study sample. Given that Australian general practice guidelines suggest biennial BMI measurements [49] in all adults aged ≥18 years and Canadian guidelines every 1 to 3 years, further research is required to confirm whether BMI data may be more complete if extracted over a 2-year (Australia) or 3-year (Canada) period as opposed to 1 year prebaseline. Furthermore, some of the missing BMI values in both datasets may be due to the recording of this information in free-text fields that are not currently extracted by CPCSSN or NPS MedicineWise (ie clinical progress notes). While there are suggested content standards for Canadian and Australian EHRs, these are not mandatory and thus, variation in data structures, formats, and content continues to exist between the many EHR products available in both these countries.

Missing dates of diagnoses were less than 10% for comorbidities that commonly cooccur with osteoarthritis, except for anxiety in the Australian EHR data. It is unknown exactly why close to 20% (35,644/201,462) of records with an anxiety diagnosis in the Australian data had a missing diagnosis onset date. Some of this may be explained by patients having long-standing anxiety, including undiagnosed anxiety, making it difficult for patients to recall the date of onset.

The prevalence of osteoarthritis was similar in the Canadian EHRs compared with national estimates from the CCDSS, except for the >80-year age group, where the prevalence was smaller in the EHRs. There were some differences in how osteoarthritis cases were defined in each data source (multiple elements within the EHR vs hospital and billing records in the CCDSS), and the addition of hospital records in the CCDSS may have accounted for older patients with more recent diagnoses of osteoarthritis that have not been documented in their general practice EHR but have undergone surgery.

Finally, the rates of TKR recorded in both the Australian and Canadian EHRs were markedly smaller than national estimates. In both countries, for a surgery such as TKR to be recorded in a patient’s general practice EHR, the patient must either inform their general practitioner of the surgery and date, or the general practice clinic receives documentation from the hospital notifying them of the surgery and date, and the general practitioner then enters this information into the EHR system. Typically, the documentation from the hospital is stored in the EHR but is not necessarily entered as a procedure in the EHR which may explain the underrepresentation of TKR surgery in this study cohort. Data relating to knee replacement surgery in Australian and Canadian general practice EHRs are likely to be unsuitable for use in osteoarthritis research in their current state. Data linkage with national joint replacement registries or hospital databases may be required if researchers wish to conduct osteoarthritis studies where the true rates of knee replacement in patients with osteoarthritis attending general practice is of interest.

### Strengths and Limitations

This study contributes to the limited literature on the quality of data in general practice EHRs. It is the first study, to our knowledge, to compare the quality of these data internationally and in the context of a globally important chronic disease, osteoarthritis. This study provides insight into specific data fields in EHRs that can be targeted for more complete recording in general practice or potential data fields for the development and testing of novel missing data methods. Furthermore, the data quality assessment methods used in this study were based on established data quality assessment guidelines [15]. Study cohort sizes were large in both datasets and therefore likely to provide a true representation of data recorded in general practice EHRs for patients with osteoarthritis. Finally, this data quality assessment was conducted with input from epidemiologists, biostatisticians, and general practitioners, and considers the context in which these data were collected.

Due to limited national datasets of patients with osteoarthritis available for data linkage in both countries, accuracy of the EHR data were unable to be assessed. Furthermore, there were differences in the dates of data extraction between the Australian and Canadian EHR datasets, with the latter containing more recent patient EHR data. While this may seem problematic at first, we applied a consistent study timeline (ie, 4-year follow-up period) and it is unlikely that recording practices relating to osteoarthritis in the Australian EHRs changed significantly between 2013 and 2017 to 2015 to 2019. There were also slight differences in the coding of osteoarthritis prescriptions between the Australian and Canadian EHR datasets due to limitations in the prescription data fields available for analysis in the Canadian dataset. This may explain the relatively higher amounts of missing data for osteoarthritis prescriptions in the Australian dataset, where missing data in any of the medication strength, dosage, or frequency fields would result in missing data for that particular osteoarthritis medication. In the Canadian dataset, missing osteoarthritis prescription data arose from missing medication codes and missing associated start dates only.

Furthermore, data quality may have impacted the study population due to inclusion and exclusion criteria. A patient was included in the study population based on the condition of having at least one encounter within prebaseline period. This could potentially artificially select for more complete EHR records and thus indicate a higher quality of data than what is available in the data sources that were under investigation for this study.

There were differences in the prescribing of anti-inflammatory and antirheumatic products (M01) and psychoanaleptics (N06) in the Australian and Canadian EHR datasets, with higher rates reported in the Canadian EHR data. In both countries, recording of over-the-counter medications in general practice EHRs requires the patient to recall this information, inform their general practitioner, and the general practitioner to record this information in the designated area of the EHR. It is possible that these medications are not captured well in Australian EHRs or are being entered elsewhere in the EHR. Reasons for the low rates of recording of N06 medications in the Australian cohort remains unknown and warrant further investigation.

Due to limited national data available on patients with osteoarthritis in both countries, we were unable to externally validate all EHR data fields of interest in this study. However, from previous work conducted by NPS MedicineWise [26] and our research team on the Australian EHR dataset [17], external validity was assessed for the recording of osteoarthritis prevalence, remoteness areas, BMI, comorbidities (hypertension, lipid disorder, ischemic heart disease, asthma, diabetes, chronic obstructive pulmonary disease, metastatic solid tumor, depression, and anxiety), and prescribing of osteoarthritis medications through comparison with the 2014 to 2015 National Health Survey. The assessment demonstrated good external validity for these data fields except for the prescribing of osteoarthritis medications (Australian EHRs 34% vs National Health Survey estimate 55%) and metastatic solid tumor (Australian EHRs 17% vs National Health Survey estimate 26%). The Australian National Health Survey asks participants to report all medication use, including over-the-counter medications and medications prescribed by specialists. These may not be captured in general practice EHRs and may explain differences in osteoarthritis medication rates between the Australian EHR data and Australian National Health Survey estimates. Further work is needed to externally validate the prescribing rates of osteoarthritis medications reported in this study. The lower rates of recorded metastatic tumors in Australian general practice EHRs may be due to tumor diagnoses by specialists not being communicated to the general practitioner. Hence, data relating to metastatic tumors in the Australian EHRs may not be fit for use in research.

Finally, the usability of these data for research is worth noting. It takes a significant amount of time to adequately clean and prepare EHR data for analysis, including quality assessments [32,50]. Text-heavy fields, such as prescription and diagnosis data, often contained typographical errors and required advanced pattern-matching searches to identify certain conditions or medications. Extensive data cleaning and preprocessing was conducted on both the Australian and Canadian datasets before assessing data quality in this study. Researchers wanting to use these data for research purposes should be made aware of the effort required to code and prepare general practice EHR data for research use. This work also highlights the strong need for better standardization of general practice EHR software systems and development of natural language processing software specific to general practice EHR data. Furthermore, conducting EHR data analysis can be a lengthy process for many reasons that may result in outdated findings; this includes challenges related to extended data governance and extraction timelines, securing funding for data access costs, and extensive data cleaning efforts.

### Conclusions

This study compared the quality of data in Australian and Canadian general practice EHRs in the context of osteoarthritis for the purposes of validating a clinical prediction model. Overall, data quality was similar in the 2 datasets. Missing and implausible data were minimal except for the recording of weight, height, BMI, and Canadian Index of Multiple Deprivation. These data fields may not be fit for use in osteoarthritis research due to large proportions of missing data that are unlikely to be recoverable using imputation techniques. External validity of recording of knee surgery in both the Australian and Canadian EHRs was poor. Better integration of patient data across primary and tertiary care is required if these data are to be used in osteoarthritis research. In the meantime, data linkage with national joint replacement and surgical registries may overcome some of these data quality challenges.

## Appendix

Multimedia Appendix 1 Coding for Australian electronic health record data.

Multimedia Appendix 2 Coding for Canadian electronic health record data.

## Abbreviations

CCDSS: Canadian Chronic Disease Surveillance System
CPCSSN: Canadian Primary Care Sentinel Surveillance Network
EHR: electronic health record
NPS: National Prescribing Service
TKR: total knee replacement

## Data Availability

The datasets analyzed during this study are not publicly available due to patient privacy concerns but may be available from the corresponding author on reasonable request if permission is granted by NPS MedicineWise, the Australian Bureau of Statistics, and the Canadian Primary Care Sentinel Surveillance Network.
