# Supplementary material for: Comparing the Quality of Primary Care Electronic Health Record Data in Australia and Canada: Case Study in Osteoarthritis
Source: J Med Internet Res. 2025 Jul 3;27:e69631. doi: 10.2196/69631 (PMC12271963; doi:10.2196/69631)
Supplement: Multimedia Appendix 2 [file jmir_v27i1e69631_app2.docx]

# Multimedia Appendix 2: Coding for Canadian EHR data

| **CONDITION** | **DEFINITION** | **VALIDITY (95% CI)** |
| --- | --- | --- |
| **OSTEOARTHRITIS** (1) | CPCSSN Definition:   - Any occurrence of ICD9 codes in billing or problem list (715, 721) | Sensitivity: 77.8% (74.5-81.1)  Specificity: 94.9% (93.8-96.1)  PPV: 87.7% (84.9-90.5)  NPV: 90.2% (88.7-91.8) |
| **HYPERTENSION** (1) | CPCSSN Definition:   - Any occurrence of ICD9 codes (401, 402, 403, 404, 405) in problem list OR - At least 2 ICD9 codes (401, 402, 403, 404, 405) in a two-year period in billing OR - Medication (ATC codes C02*, C03AA03, C03BA04, C03BA08, C03BA11, C03DB01, C03DB02, C03EA01, C07AA06, C07AB03, C07AB04, C07AG02, C07CB03, C08CA01, C08CA02, C08DA01, C09AA01, C09AA02, C09AA03, C09AA07, C09AA08, C09AA09, C09AA10, C09BA02, C09BA03, C09CA02, C09CA03, C09CA04, C09CA07, C09DA01, C09DA02, C09DA04, C09XA02) *unless* ICD9 codes exist (250, 333.1, 346, 410, 412, 413, 427, 428, 456.0, 456.1, 592, 572.3) | Sensitivity: 84.9% (82.6-87.1)  Specificity: 93.5% (92.0-95.1)  PPV: 92.9% (91.2-94.6)  NPV: 86.0% (83.9-88.2) |
| **LIPID DISORDER** (2) | Definition validated on one regional CPCSSN network:   - At least one lab value outside normal range (total cholesterol >5.2 mmol/L, HDL-C < 1.0 mmol/L, LDL-C > 3.4 mmol/L, triglycerides >1.7 mmol/L) OR - History of a lipid modifying agent (ATC code C10*) within 2 years before date of lipid test | Sensitivity: 100%  Specificity: 100%  PPV: 100%  NPV: 98% |
| **ISCHAEMIC HEART DISEASE** | ICD9 410-414;  *CPCSSN definition for CAD in development* | n/a |
| **DEPRESSION** (1) | CPCSSN Definition:   - Any occurrence of ICD9 codes in billing or problem list (296, 311) OR - Medication (ATC code N06AB*, N06AF04, N06AG02, N06AX11, N06CA01 *unless* ICD9 300 (anxiety) is present | Sensitivity: 81.1% (77.2-85.0)  Specificity: 94.8% (93.7-95.9)  PPV: 79.6% (75.7-83.6)  NPV: 95.2% (94.1-96.3) |
| **Anxiety and other neurotic, stress related and somatoform disorders** | ICD9 300, 308, 309;  *CPCSSN definition in development* | n/a |
| **ASTHMA** | ICD9 493;  *CPCSSN definition in publication (Cave et al)* |  |
| **DIABETES MELLITUS** (1) | CPCSSN Definition:   - Any occurrence of ICD9 code (250) in problem list OR - Minimum 2 occurrences of ICD9 code (250) within 2 years OR - HbA1C ≥7% OR at least 2 occurrences within 1 year of fasting glucose >7% OR - Medication (ATC code A10*) *unless* ICD9 249, 256.4, 648.8, 775.1, 790.29 is present | Sensitivity: 95.6% (93.4-97.9)  Specificity: 97.1% (96.3-97.9)  PPV: 87.0% (83.5-90.5)  NPV: 99.1% (98.6-99.6) |
| **CHRONIC OBSTRUCTIVE PULMONARY DISEASE (COPD)** (1) | CPCSSN Definition:   - Patient age ≥35 AND - Any occurrence of ICD9 codes in billing or problem list (491.2, 492, 496) OR - Medication (ATC codes R03BB*, R03AK04, R03AL*) *unless* ICD9 493 (asthma) is present | Sensitivity: 82.1% (76.0-88.2)  Specificity: 97.3% (96.5-98.0)  PPV: 72.1% (65.4-78.8)  NPV: 98.4% (97.9-99.0) |
| **METASTATIC SOLID TUMOUR** (3) | ICD9 196.x-199.1  *No CPCSSN definition available* |  |
| **Count of chronic conditions from the Charlson Comorbidity Index** | | |
| The R package “charlson_icd9_quan” was used to code Charlson comorbidities from ICD-9 data (3) | | |

### CI=confidence interval; PPV=positive predictive value; NPV=negative predictive value

1. Williamson T, Green ME, Birtwhistle R, Khan S, Garies S, Wong ST, et al. Validating the 8 CPCSSN case definitions for chronic disease surveillance in a primary care database of electronic health records. Ann Fam Med [Internet]. 2014;12(4):367–72. Available from: https://pubmed.ncbi.nlm.nih.gov/25024246/

2. Aref-Eshghi E, Oake J, Godwin M, Aubrey-Bassler K, Duke P, Mahdavian M, et al. Identification of Dyslipidemic Patients Attending Primary Care Clinics Using Electronic Medical Record (EMR) Data from the Canadian Primary Care Sentinel Surveillance Network (CPCSSN) Database. J Med Syst [Internet]. 2017;41(3):45. Available from: https://pubmed.ncbi.nlm.nih.gov/28188559/

3. Quan H, Sundararajan V, Halfon P, Fong A, Burnand B, Luthi JC, et al. Coding algorithms for defining comorbidities in ICD-9-CM and ICD-10 administrative data. Med Care [Internet]. 2005 Nov;43(11):1130–9. Available from: https://pubmed.ncbi.nlm.nih.gov/16224307/
